# An Endotenon Sheath-Inspired Double-Network Binder Enables Superior Cycling Performance of Silicon Electrodes

**DOI:** 10.1007/s40820-022-00833-5

**Published:** 2022-04-01

**Authors:** Meifang Jiang, Pengzhou Mu, Huanrui Zhang, Tiantian Dong, Ben Tang, Huayu Qiu, Zhou Chen, Guanglei Cui

**Affiliations:** 1 Qingdao Industrial Energy Storage Research Institute, Qingdao Institute of Bioenergy and Bioprocess Technology, Chinese Academy of Sciences, No. 189 Songling Road, Qingdao, 266101 People’s Republic of China; 2 Key Laboratory of Marine Chemistry Theory and Technology Ministry of Education, College of Chemistry and Chemical Engineering, Ocean University of China, No. 238 Songling Road, Qingdao, 266100 People’s Republic of China; 3 University of Chinese Academy of Sciences, Beijing, 100190 People’s Republic of China

**Keywords:** Efficiency, Double-network binder, Silicon electrodes, Lithium battery

## Abstract

Silicon (Si) has been regarded as an alternative anode material to traditional graphite owing to its higher theoretical capacity (4200 vs. 372 mAh g^−1^). However, Si anodes suffer from the inherent volume expansion and unstable solid electrolyte interphase, thus experiencing fast capacity decay, which hinders their commercial application. To address this, herein, an endotenon sheath-inspired water-soluble double-network binder (DNB) is presented for resolving the bottleneck of Si anodes. The as-developed binder shows excellent adhesion, high mechanical properties, and a considerable self-healing capability mainly benefited by its supramolecular hybrid network. Apart from these advantages, this binder also induces a Li_3_N/LiF-rich solid electrolyte interface layer, contributing to a superior cycle stability of Si electrodes. As expected, the DNB can achieve mechanically more stable Si electrodes than traditional polyacrylic acid and pectin binders. As a result, DNB delivers superior electrochemical performance of Si/Li half cells and LiNi_0.8_Co_0.1_Mn_0.1_O_2_/Si full cells, even with a high loading of Si electrode, to traditional polyacrylic acid and pectin binders. The bioinspired binder design provides a promising route to achieve long-life Si anode-assembled lithium batteries.

**Supplementary Information:**

The online version contains supplementary material available at 10.1007/s40820-022-00833-5.

## Highlights

- The double-network binder shows excellent adhesive and self-healing abilities, which help suppress electrode volume expansion and stabilize the electrode interface upon cycling.
- This binder induces a Li_3_N/LiF-rich solid electrolyte interface layer, which can suppress continuous electrolyte decomposition.
- Superior electrochemical performance can be achieved in Si/Li half cells and LiNi_0.8_Co_0.1_Mn_0.1_O_2_/Si full cells, even with a high loading of Si electrode.

**Supplementary Information:**

The online version contains supplementary material available at 10.1007/s40820-022-00833-5.

## Introduction

To meet the ever-growing energy density requirements for future electrical energy storage and all-electric vehicle applications, high specific capacity electrode materials are urgently needed. Silicon (Si), as one of the most promising anode materials, has been attracting significant attention owing to its high theoretical capacity (4200 mAh g^−1^) far exceeding that of graphite anodes (372 mAh g^−1^). Additionally, silicon anodes have the advantages of low operating voltages (0.1–0.4 V vs. Li^+^/Li), environmental benignity, and vast natural abundance. Nonetheless, there remain severe challenges for practical applications of Si anodes. The main problem involves rapid battery capacity fade owing to the dramatic volume expansion of Si (~ 300%) during full lithiation, which often results in pulverization of Si particles, loss of electrical contact between active materials and the current collector, and the incompatible solid electrolyte interphase (SEI) [1, 2].

In order to address the problem confronting Si anodes, existing approaches include structural modifications of Si materials (e.g., nanowires, nanosheets and hierarchical constructions), introduction of additives and the development of novel binders [3–8]. Among these strategies, the employment of advanced binders has been demonstrated to be a facile yet effective way without compromise in battery energy density [9, 10]. Ideal binders should tolerate the volume expansion of Si particles upon cycling by relying on the reversibility of their bonding structure, thereby maintaining structural integrity of Si electrodes [11, 12]. To date, many synthetic and natural polymers with either linear or cross-linked polymer backbone have been explored and applied as promising binders for Si anodes, such as poly(acrylic acid) (PAA) [13], sodium carboxymethyl cellulose (CMC) [14], sodium alginate (Alg) [15], blend polymers (i.e., CMC/PAA) [16], and so on. These binders can improve cycling stability of the as-assembled batteries to some extent; however, they are rarely robust enough to realize mechanically stable Si electrodes upon cycling. This is attributable to the fact that existing binders including so-called self-healing type ones can hardly ensure stable cycling of Si electrodes: (1) Although the self-healing ability of polymeric bonding networks with supramolecular interactions (e.g., hydrogen-bonding and ion–dipole interactions) can facilitate the recovery of electrode films during delithiation, it hardly restores to the original state (i.e., the bonding location) before last lithiation; the incomplete recovery of bonding locations is highly correlated with the mechanical mismatch between Si particles and binders. (2) Huge volume change of Si electrodes upon lithiation/delithiation undoubtedly results in, in part, the fracture of SEI; this exposes fresh contact between active materials and electrolytes, leading to continuous electrolyte decomposition and thus fast battery capacity decay together with low Coulombic efficiency. Therefore, developing a functional binder to achieve stable cycling of Si electrodes continues to be highly sought.

**Fig. 1:**
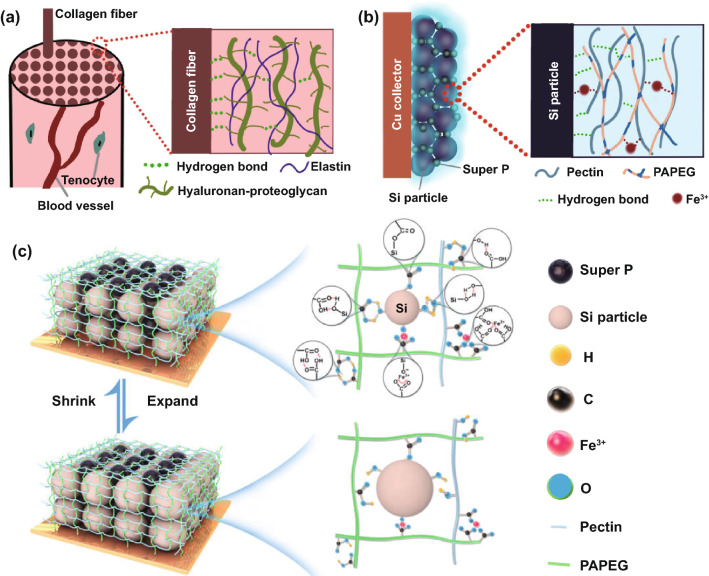
Schematic illustration of **a** endotenon sheath in tendon, **b** bioinspired double-network polymer binder design, and **c** the supramolecular hybrid network in Si electrodes during cycling

To address the above-mentioned issues, herein, we report a bioinspired tough double-network binder (DNB) for Si electrodes. The design of the DNB is inspired by the endotenon sheath of tendon in the human body [17]. The endotenon sheath is mechanically robust, which shows strong adhesion with the collagen fibers, tenocytes, and blood vessels (Fig. 1a). The most attractive point for this kind of adhesion is that it can achieve wonderful mechanical match (i.e., accommodating volume deformation and withstanding external stress) and perfect bonding recovery during exercise. These benefits are ascribed to its unique double-network polymer structure: The high-viscosity hyaluronan–proteoglycan strongly binds with the collagen fibers via supramolecular interactions, while the elastin with both the hydrophilic and oleophilic segments strengthens the adhesion structure by forming supramolecular interactions with hyaluronan–proteoglycan. Learning from the structure and function of the endotenon sheath, we propose a mechanically robust DNB (design strategy illustrated in Fig. 1b) for Si electrodes, which contains pectin with high viscosity and the amphipathic copolymer PAPEG comprised of hydrophilic polyacrylic acid (PAA) and oleophilic polyethylene glycol diacrylate (PEGDA). In addition, the ferric nitrate [Fe(NO_3_)_3_] is further introduced to construct coordinate bonds among carboxylic acid units on the polymers for dissipating stress. The DNB not only can strongly glue Si particles by forming hydrogen bonding, but also can form supramolecular interactions (e.g., hydrogen-bonding and ion–dipole interactions) between pectin and PAPEG to strengthen adhesion structure (Fig. 1c). The formed supramolecular hybrid network of DNB helps stabilize the electrode interface when experiencing volume shrinkage and expansion of Si electrode upon cycling and reduce electrolyte decomposition originating from its mechanical damage. Moreover, ferric nitrate can be reduced into Li_3_N contributing to constructing compatible SEI. Both of merits are expected to jointly achieve stable cycling of Si electrodes.

Different from the design of most previously reported binders, our approach features a tough double-network polymer backbone with a supramolecular hybrid network (e.g., hydrogen-bonding and ion–dipole interactions), which renders an excellent self-healing capability. Furthermore, it is demonstrated that the as-developed binder can help form a Li_3_N/LiF-rich SEI layer, which can suppress continuous electrolyte decomposition. As a result, DNB renders superior electrochemical performance in Si/Li half cells and LiNi_0.8_Co_0.1_Mn_0.1_O_2_ (NCM811)/Si full cells, even with a high loading of Si electrode. Specifically, the capacity of Si/Li half cells with DNB binders can still remain at 1115 mAh g^−1^ after 300 cycles at a current density of 4.2 A g^−1^ (1 C), much superior to traditional PAA and pectin binders. Furthermore, the as-developed binder also endows NCM811 (11 mg cm^−2^)/Si full cells with good cycle performance, with a capacity retention of 86% at 0.1 C after 50 cycles, evidently obviously exceeding that of the PAA counterpart. This design philosophy of Si electrode binders provides a promising strategy to regulate the mechanochemistry of Si electrodes.

## Experimental and Calculation

### Materials

Pectin (Mn = 41 KDa), APS (98.0%), and AA (99.7%) were purchased from Aladdin, and PAA (Mn = 450 KDa) and poly (ethylene glycol) dimethacrylate (PEGDA, Mn = 600 Da, 99.8%) were purchased from Macklin. Fe(NO_3_)_3_·9H_2_O (98%) was purchased from Alfa Aesar. Nano-Si was purchased from Quzhou Guangtong New Material Technology. The average diameter of the nano-Si is about 100 nm as shown in Fig. S1. X-ray diffraction (XRD) characterization of Si particles reveals that all peaks correspond well to standard crystallographic data (Fig. S2) [International Centre for Diffraction Data (ICDD) File No. 001-0791] [18].

### Preparation of DNB

The PAPEG polymer was synthesized via radical polymerization of polyacrylic acid (AA) and polyethylene glycol diacrylate (PEGDA) in aquatic environment and initiated by APS. Pectin contains abundant functional groups such as hydroxyl, carboxyl, and ether bonds, which will facilitate proper wetting with carbonate electrolytes. Before the polymer was synthesized, Fe(NO_3_)_3_ was added in to the precursor in a molar ratio of n(PAA)/Fe^3+^ = 200:1, and then stirring under 45 °C conditions for about 4 h.

### Preparation of Binders

PAA binder (5%) was prepared by 0.5 g PAA and 9.5 g deionized water. Pectin binder (5%) was prepared by 0.5 g pectin and 9.5 g deionized water. For DNB (5%), AA (0.7 g), pectin (0.07 g), APS (0.035 g), PEGDA (0.001 g), Fe(NO_3_)_3_·9H_2_O (0.02 g), and deionized water (15 g) were poured into the bottle and stirred at 45 °C to prepare a viscous and homogeneous solution.

### Preparation of Si Anode

The Si electrodes were prepared by mixing binder with super P and Si nanoparticles in the weight ratio of 2:2:6. The well-mixed slurry was scraped on the copper foil using a doctor blade, then drying in a vacuum oven at 80 °C for 12 h.

### Electrochemical Tests

Half-cell tests were assembled in a CR 2032 type coin cell, the Li metal as the counter electrode, 1 M LiPF_6_ in EC/DEC (v/v 1:1) with 10 wt% fluoroethylene carbonate (FEC) additive as liquid electrolyte, a polypropylene microporous film (Celgard 2400) as the separator. The electrolyte/silicon ratio was controlled at 20 μL mg^−1^. To assemble full cells, LiNi_0.8_Co_0.1_Mn_0.1_O_2_ (NCM811) electrode consisting of NCM811, super P, and polyvinylidene fluoride (PVDF) in a weight ratio of 8:1:1 was used as the cathode. The Si anodes were pre-charged and pre-discharged for three cycles at a current density of 0.1 C. The pre-cycled Si electrodes with a mass loading of 0.7 mg cm^−2^ were paired with NCM 811 cathodes (mass loading of 11 mg cm^−2^) with an N/P ratio of 1.2. The full cell was cycled between 3.0 and 4.2 V at a small current density of 0.1 C and room temperature. The electrochemical impedance spectroscopy (EIS) was measured with the applied frequency from 7 to 100 mHz with the amplitude of 10 mV. Cyclic voltammograms (CV) measurements were conducted between 0.001 and 1.5 V at a scan rate of 0.1 mV s^−1^.

### Sample Characterization

The adhesion strength of different binders to Cu current collector was conducted by a universal test machine (MTS, E43). The electrode films were cut into rectangular shapes with 2 × 7 cm^2^ after drying in vacuum oven for 12 h. The active material side of electrode was adhered to a wood bar using 3 M double-sided adhesive tape, and the Cu current collector side was adhered using traditional 3 M tape. The end of the 3 M tape is fixed to the machine, and then, the tape is peeled off at a constant traction speed. The value of the peeling strength changing with time can be obtained. Tensile experiments of polymer binders were performed under ambient condition. For the tensile tests, the dry polymer films were cut into strip. The tensile tests were carried out at a strain rate of 5 mm min^−1^ using a Universal testing machine. XRD patterns were recorded in a Bruker-AXS Micro-diffractometer (D8 ADVANCE) with Cu-K_α1_ radiation (*λ* = 1.5405 Å). The in situ optical microscope (Cossim CMY-400Z optical microscope) was used to observe the cross section of Si electrode with different binder in real time in order to study the electrode deformation process during cycling. The Fourier-transform infrared spectrum (FT-IR, Bruker VERTEX 70) was used to investigate the chemical structures of samples. The morphology of samples was characterized by scanning electron microscope (SEM Hitachi S-4800). X-ray photoelectron spectroscopy (XPS) analysis was carried out using PerkinElmer PHI 550 spectrometer equipped with Ar^+^ sputtering gum using Al Kα source. The rheology experiment was performed at 17 °C by the ARES-G2 to identify the shear viscosity and modulus of these binder solution (5 wt%) at different angular frequency. Nanoindentation was conducted using Nanotest Vantage. Contact angles of LiPF_6_/EC-DEC-FEC electrolyte on different films were conducted using Theta Flex.

### Calculation Details

All the spin-polarized density functional theory (DFT) calculations were implemented by Vienna ab initio simulation package (VASP 5.4.4) based on plane-wave DFT [19]. Generalized gradient approximation (GGA) of PBE was performed [20], and the core–valence electron interaction was treated by the projector-augmented, with the valence electrons: 1*s*^1^ for H, 2*s*^2^2*p*^4^ for O, 2*s*^2^2*p*^2^ for C, 3*s*^2^3*p*^2^ for Si, and 3*d*^6^4*s*^2^ for Fe [21]. The convergence criteria for the electronic minimization are set at the energy difference of 10^–5^ eV. Relaxation of atomic positions was stopped when all forces are less than 0.02 eV Å^−1^. Cutoff energy of 500 eV was chosen, which is high enough to have the total energy converged at one meV atom^−1^.

To describe the interactions between the binder and Si particles, a 3 × 3 Si unit cell was selected with the two floors in the bottom fixed. The vacuum layer was set to 20 Å. For geometry optimization, k-points of (2 × 2 × 1) in the reciprocal space of the Monkhorst–Pack scheme were chosen to ensure convergence [22], and the Gaussian smearing with an energy of 0.1 eV was used for the electronic occupancies. The following equation calculates the ${E}_{\mathrm{ads}}$ of the binder and surface of Si.

1 $$E_{{{\text{ads}}}} = E_{{\text{substrate + molecule}}} - E_{{{\text{substrate}}}} - E_{{{\text{molecule}}}}$$

where $E_{{\text{substrate + molecule}}}$ is the total energy of the system, $E_{{{\text{substrate}}}}$ is the total energy of the clean substrate, and $E_{{{\text{molecule}}}}$ is the energy of the isolated molecules. Fe atom are calculated in an isolated cell. For ferric ions-containing fragments over the Si surface, ${E}_{\mathrm{molecule}}$ is disassembled into two parts to calculate formation energy, including ${E}_{{\mathrm{Fe}}^{3+}}$ and $E_{{\text{substrate + molecule}}}$.

## Results and Discussion

### Preparation and Characterizations of the As-Investigated Binder

The water-soluble DNB is prepared by mixing commercially available pectin with Fe(NO_3_)_3_·9H_2_O, and the preprepared PAPEG at 40 °C (Experimental section). The electrochemical stability of the DNB was measured through cyclic voltammetry (CV). In this test, the electrode obtained by gluing super P onto a Cu current collector with DNB was used as the working electrode, which was operated at a voltage range of 0.01–1.5 V versus Li^+^/Li. As can be seen in Fig. S3, during the first cycle, the CV profile exhibits two cathodic peaks at around 1.6 and 0.7 V. The distinct reduction slope from 1.6 to 1.0 V during the first cathodic scan can be attributed to the reduction of NO_3_^−^ in DNB. Meanwhile, the cathodic peak at around 0.7 V corresponds to the reduction of carbonate solvents [23]. In the subsequent cycles, the CV profile does not show any noticeable redox peaks, demonstrating the good electrochemical stability of DNB. Furthermore, the Fe 2p XPS spectra of Si electrodes before and after 30 cycles demonstrate that there is no obvious reduction of Fe^3+^ from DNB (Fig. S4), further supporting this point.

To realize the effect of the as-developed binder on the ionic transport kinetics of Si electrodes, we examined the binder wettability by carbonate liquid electrolyte, which directly influences the permeability of electrolyte within the electrode. It is demonstrated that there is a smaller contact angle 18.00° between DNB film and carbonate electrolyte than those of the PAA (28.78°) and pectin films (31.29°) (Fig. S5). This finding indicates the better wettability of the DNB, which will facilitate Li-ion diffusion within Si electrodes.

### Supramolecular Hybrid Network and Property Characterizations of DNB

**Fig. 2:**
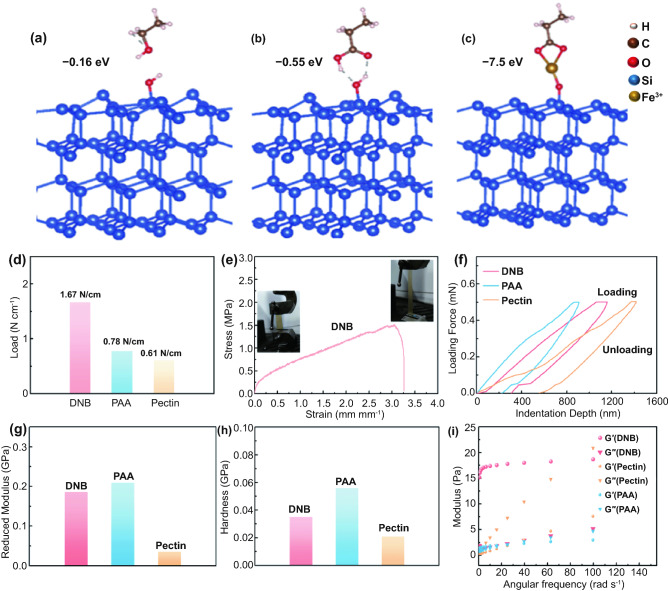
Adsorption configuration for **a** hydroxy-containing, **b** carboxyl-containing, and **c** ferric ions-containing fragments over the amorphous Si surface. White, brown, red, blue, and yellow spheres represent H, C, O, Si, and Fe^3+^, respectively. **d** Adhesion strength between Cu current collectors with different binders measured by the peel test. **e** Strain–stress curves of the DNB. **f** Comparison of load–indentation depth curves of electrodes with different binders. **g** Reduced modulus and **h** hardness of Si anodes with different binders obtained from nanoindentation tests. **i** Rheology of DNB, PAA, and pectin solutions in the angular frequency range from 0 to 100 rad/s (G′: elastic modulus, G′′: viscous modulus)

The supramolecular hybrid network within DNB is investigated by FTIR spectrum comparison of Si particles, DNB, and the composite comprised of Si particles and DNB (denoted as Si-DNB) (Fig. S6). It is evident that the addition of Si particles results in the intense and shift change of the –OH peak in the DNB, from the two strong peaks located at 3488 and 3357 cm^−1^ to a weak peak at 3305 cm^−1^. This observation suggests that abundant hydrogen bonds are formed between DNB and Si particles. On the basis, we simulated the adsorption configuration through first-principle calculations to describe the interactions between the binder and Si particles. To simplify the model, the DNB is divided into three parts: hydroxy-containing (Fig. 2a), carboxyl-containing (Fig. 2b), and Fe ions-containing fragments (Fig. 2c). It is demonstrated that these fragments interact with the surface oxygen species of amorphous Si with adsorption energies of − 0.16, − 0.55, and − 7.5 eV, respectively. These results imply that there are spontaneous H-bonding and ion–dipole interactions between the DNB and Si particles.

Additionally, the introduction of Fe^3+^ is conductive to constructing the ion–dipole interaction between Fe^3+^ and DNB. Carboxylate groups in DNB show two IR characteristic absorbance bands: One is the asymmetric vibration (*ʋ*_asym_) located at the higher frequencies, and the other is symmetric vibration (*ʋ*_sym_) at the lower frequencies. The separation (Δ*ʋ*) between *ʋ*_asym_ and *ʋ*_sym_ of carboxylate in DNB is about 172 cm^−1^, while the one in the DNB analogue without Fe(NO_3_)_3_ (denoted as PAPEG-pectin) is about 220 cm^−1^ (Fig. S7a). The decreased Δ*ʋ* indicates that the coordination between metal ion and carboxylate groups occurs in the DNB [24, 25]. Furthermore, after the introduction of Fe^3+^, the *ʋ* (OH) of pectin from DNB splits into two peaks located at around 3487.7 and 3356.5 cm^−1^ (Fig. S7b), demonstrating the coordination between Fe^3+^ and − OH groups. Thus, a dynamic cross-linking framework can be formed via the ion–dipole or hydrogen-bonding interaction among Fe^3+^ and functional groups (i.e., carboxylate acid and hydroxyl groups) in DNB. The supramolecular hybrid network formed by DNB can be mirrored by the high viscosity of the electrode slurry evidenced by the rheology test. By identifying the dynamic viscosities of binder solutions at different angular frequency from 1 to 300 rad s^−1^, we observe that the electrode slurry with the DNB displays an obviously higher viscosity after the introduction of Fe^3+^ (Fig. S8). The supramolecular hybrid network within DNB-based Si electrodes is expected not only to render a robust adhesion of Si electrodes, but also to incur a high self-healing ability of Si electrodes.

It is investigated that DNB renders excellent adhesive ability of Si electrodes, as quantitatively evaluated by the peeling test. As seen from Figs. 2d and S9, DNB shows a high adhesion strength value of 1.67 N cm^−1^, almost 2 times higher than that of PAA (0.78 N cm^−1^) and pectin (0.61 N cm^−1^) binders. This finding can be supported by the stripping extent of active materials on Si electrode. As shown in Fig. S10, Si electrodes with DNB show the least stripping of active materials than the PAA and pectin counterparts after peeling tests. Additional proof for the superior adhesion of DNB is that it can enable repeated fold and adhere on Cu current collector, in sharp contrast with the obvious active material stripping of PAA and pectin binders **(**Fig. S11). These results verify that the DNB shows excellent adhesive ability with electrode materials and Cu current collector, mainly ascribed to the formation of the reversible supramolecular hybrid network. The strong adhesion of DNB is valuable for maintaining a robust ionic/electronic conductive network within Si electrodes during cell cycling.

DNB films and the corresponding Si electrode also exhibit excellent mechanical properties. The DNB can endure approximately 350% stretching and withstand stress up to about 1.5 MPa, as shown in Fig. 2e. These admirable mechanical properties of the DNB can arise from its special double-network structure and high self-healing ability. In addition, the superior mechanical properties of DNB-based electrodes are also confirmed by a nanoindentation technology. The typical load–displacement curves of the electrodes with different binders are shown in Fig. 2f. Under the same force of 0.5 mN, the indentation depth on DNB-based Si electrodes is deeper than that of the PAA-based electrode, but is shallower than the electrode with pectin binder counterpart. When the loading is removed, the residual indentation on the pectin-based Si electrode is deepest, followed by DNB-based Si electrode. The corresponding reduced modulus and hardness for Si electrodes with different binders are shown in Fig. 2g and h, respectively. It is evident that the DNB-based Si electrode delivers mediocre reduced modulus and hardness among electrodes with these binders. This finding implies that DNB binder not only can suppress the excessive volume expansion of Si electrodes during cycling by a decent mechanical strength, but also can dissipate stress from volume change of Si particles and realize self-healing of electrode films; these behaviors help to achieve stable cycling of Si electrodes. In comparison, PAA-based Si electrodes that show the highest reduced modulus and hardness are too rigid and brittle to recover the pristine bonding location during cycling, while pectin-based Si electrodes with the lowest reduced modulus and hardness mean much easier deformation of electrodes during cycling, which leads to more severe electrolyte decomposition. Therefore, the rigid–flexible coupling mechanical properties of the DNB are conducive to realizing stable cycling performance of Si anodes [26].

The supramolecular hybrid network of DNB allows the as-developed electrode films to deliver good self-healing abilities. Rheological investigation demonstrates that DNB shows a higher elastic modulus (G′) than the corresponding viscous modulus (G″) and keeps stable G′ and G″ in the range of 10^−1^–10^2^ rad s^−1^ (Fig. 2i). This result implies that DNB behaves like a solid-like material and meanwhile shows good self-healing behaviors by virtue of its reversible supramolecular hybrid network [27, 28]. However, PAA binder exhibits a transition behavior from solid to fluid-like with angular frequency increase, indicating that its network structure is deformed at high angular frequencies, while pectin binder delivers an elastic modulus (G′) lower than the corresponding viscous modulus G″ and meanwhile both of values continuously rise with the increase in angular frequency, showing fluid-like behaviors. There observations indicate that volume expansion of Si electrodes will more easily occur in the presence of pectin or PAA binder, compared with the DNB. To provide intuitive evidence for good self-healing ability of DNB, we cut the DNB film into two pieces and kept them together for about 4 h at room temperature. It is evident that the DNB film is obviously recovered (Fig. S12). The above-mentioned results reveal that the well-structured polymer network of DNB can deliver an excellent self-healing ability, verifying the rationality of DNB design.

### Mechanical Stability Evaluation of Si Electrodes Enabled by DNB

**Fig. 3:**
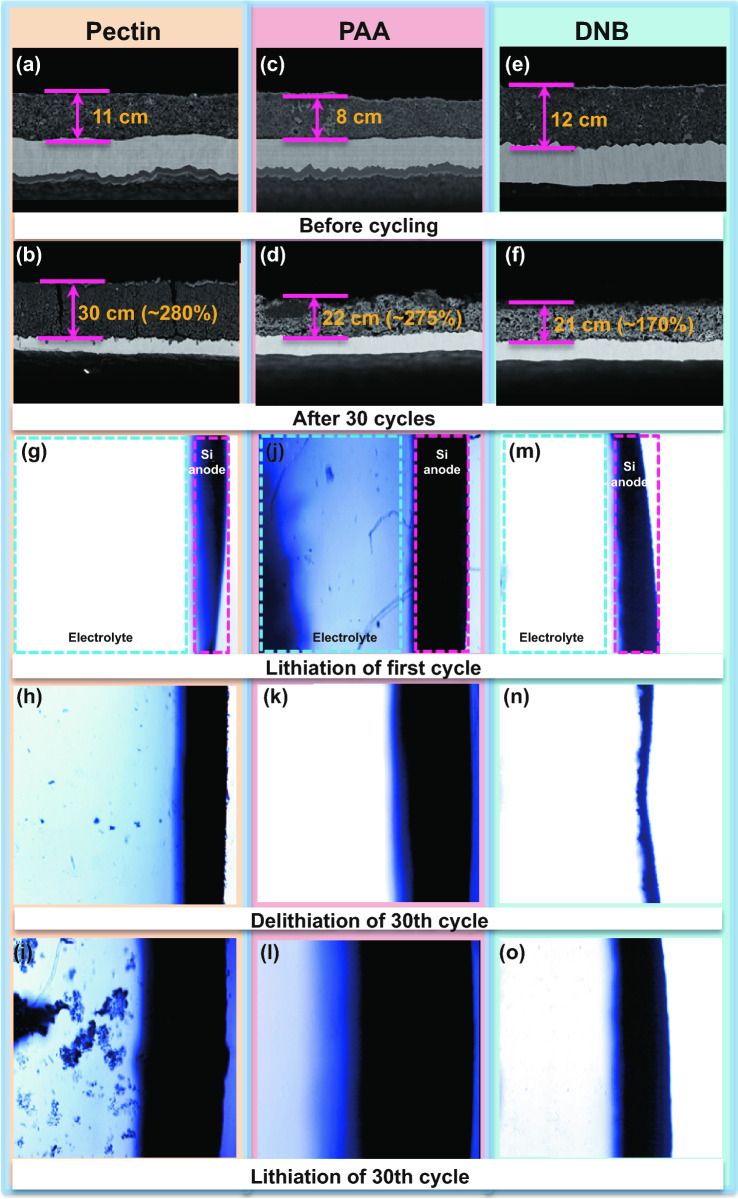
Cross-sectional SEM images of Si electrodes before (**a**–**c**) and after 30 cycles (**d**–**f**) with pectin, PAA, and DNB, respectively. In situ optical microscopy images of volume change of Si electrodes upon lithiation of first cycle and lithiation/delithiation of 30th cycle with **g**–**i** pectin binder, **j**–**l** PAA binder, and **m**–**o** DNB in assembled model cell module

To verify if DNB can improve structural stability of Si electrodes during cycling, the lithiation/delithiation behavior of as-prepared Si electrodes was evaluated. Generally, a mechanically more stable Si electrode can be mirrored by its less volume expansion after repeated cycling. Figure 3a–f summarizes the thickness variation of the Si electrodes with different binders before and after 30 cycles. The DNB-based electrode shows a thickness enhancement of ~ 170%, much smaller than those of PAA (~ 275%) and pectin (~ 280%) counterparts. These observations mean that DNB can effectively suppress the excessive volume expansion of Si electrodes during cycling. The in situ optical microscope (OM) further shows that the DNB can well maintain the integrity of the electrode structure and suppress electrode expansion during repeated cycling without active materials stripping into the electrolyte after 30 cycles (Fig. 3i–o). On the contrary, the structure of the PAA- and pectin-based Si electrodes obviously thickens and collapses severely after cycling, with good amounts of active materials falling off electrodes (Fig. 3h–n, g–m). Furthermore, a smoother surface morphology for Si electrodes with DNB without obvious cracks after 30 cycles at 0.2 C is also in favor of these findings (Fig. S13). The above-mentioned results indicate that the DNB can better achieve the deformation reversibility of Si electrodes, compared with the PAA and pectin counterparts, implying that DNB can render good structural stability of Si electrodes.

Good mechanical stability of Si electrodes can be reflected by electrochemical polarization during cycling. Figure S14 shows the CV curves of the different binders-based Si electrodes at a scanning rate of 0.1 mV s^−1^. The potential difference (Δ*E*) between the anodic and cathodic peak represents electrochemical polarization. It is evident that the DNB-based Si electrode shows the smallest ΔE, implying the lowest electrochemical polarization. Besides, the highest redox CV peaks of the Si/C electrode with the DNB mean the largest Li-ion transport kinetics in the DNB-based Si electrodes during battery operation. Furthermore, the lowest electrochemical polarization can be clearly evidenced by the lowest battery impedance during cycling among different binders (Fig. S15).

**Fig. 4:**
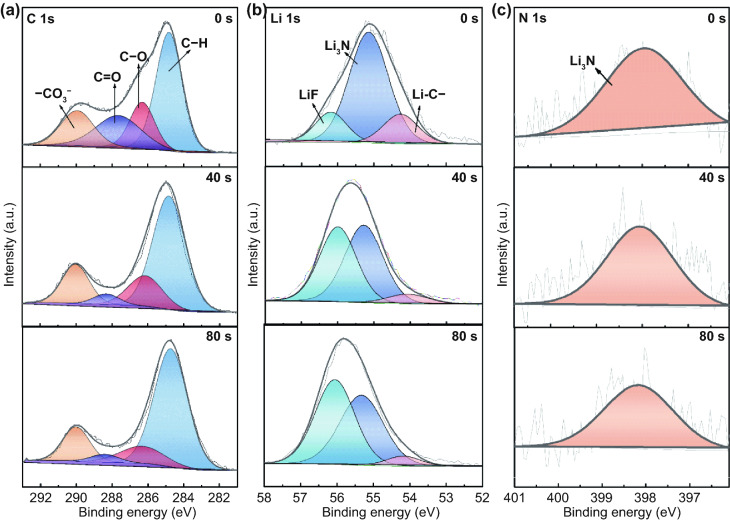
**a** C 1s, **b** Li 1s*,* and **c** N 1s XPS spectra of different Ar ion sputtering times with the DNB-based Si electrode after 30 cycles

Mechanically more stable Si electrodes can be also supported by a compatible SEI. To verify this, the thickness and chemical components of SEI on the surface of the Si particles after 30 cycles were investigated. TEM imaging shows that the thickness of SEI layer toward DNB is about 20 nm (Fig. S16). The SEI compositions formed on the cycled Si electrode with DNB were characterized via in-depth X-ray photoelectron spectroscopy (XPS) with Ar-ion sputtering (the speed of Ar-ion sputtering is about 0.25 nm s^−1^) (Fig. 4). In the C 1s spectra, the C–H (284.8 eV), C–O (286.3 eV), C=O (287.8 eV), and –CO_3_^−^ (290 eV) can be found in the upper and inner layer of SEI [29]. C 1s spectrum comparison of cycled DNB-based Si electrodes with that of cycled PAA-based, pectin-based electrodes shows that the intensity of the –CO_3_^−^ peak is stronger in PAA-based (Fig. S17), pectin-based electrodes (Fig. S18). This proves that more electrolyte decomposition side reactions that occur at the interface of the cycled PAA-based, pectin-based, and the SEI generated tend to be unstable and prone to rupture [30, 31]. In the Li 1s and N 1s spectra, there are obvious LiF (56.1 eV) and Li_3_N (55.1 eV for Li 1s spectrum, 398.2 eV for N 1s spectrum) signals [32], which mainly result from the decomposition of LiPF_6_, and Fe(NO_3_)_3_. LiF is generally regarded as an excellent SEI component for its high mechanical strength, large surface energy, and relatively small lattice constant rendering high mechanical stability and easily plastic deformation of SEI [33], while Li_3_N is a lithium super ionic conductor, which can help enhance the ion transport property of the SEI and improve the electrochemical reaction kinetics [34]. These results demonstrate that the DNB helps build up a SEI rich in Li_3_N and LiF, which is expected to deliver good mechanical stability and fast ionic transport kinetics. These observations confirm that a Li_3_N/LiF-rich SEI occurs in the cycled DNB-based Si electrode. Thus, it can be argued that the DNB helps to construct a compatible SEI, which can obviously suppress continuous electrolyte decomposition, benefited for achieving stable cycling of Si electrodes.

### Performance Evaluation of Batteries with DNB-Based Si Anodes

**Fig. 5:**
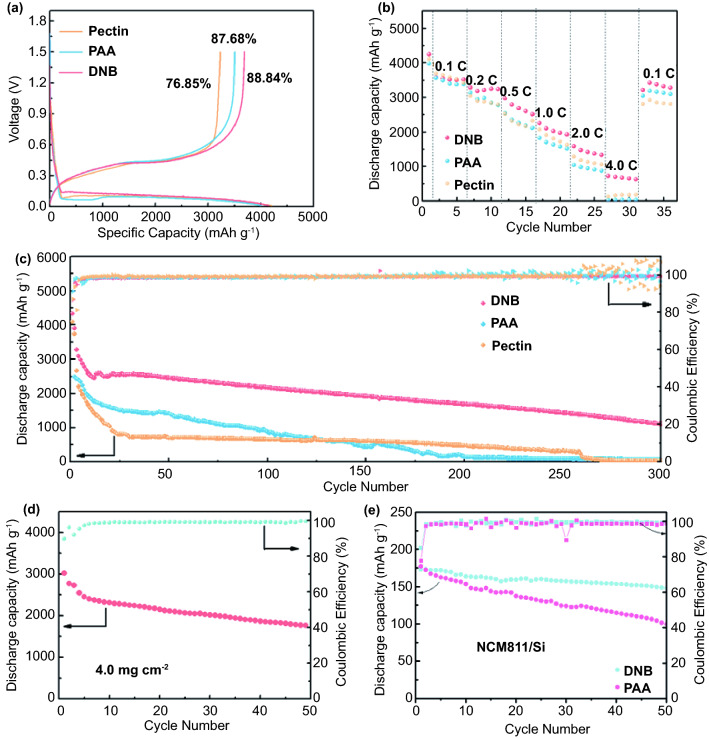
**a** The initial charge–discharge profiles of Si electrodes with different binders when measured at 0.2 C. **b** Rate capability of the Si (1.0 mg cm^−2^)/Li cells with different binders at different current rates. **c** Long-term cycling performance and Coulombic efficiency of the Si (0.78 mg cm^−2^)/Li cells with different binders at 1 C, room temperature, and a voltage range of 0.01–1.5 V. **d** Cycling performance of the Si (4.0 mg cm^−2^)/Li cells with DNB at 0.1 C, room temperature, and a voltage range of 0.01–1.5 V. **e** Cycling performance of NCM811 (~ 11.0 mg cm^−2^)/Si full cells with DNB at 0.1 C, room temperature, and a voltage range of 3.0–4.2 V

As a proof of concept, the effect of DNB on electrochemical performance of Si anodes was evaluated. Figure S19 shows the cycling performance of electrodes with different binders at a current density of 0.84 A g^−1^ (0.2 C). The DNB-based Si electrode exhibits an obviously enhanced capacity retention (56%) than those of PAA (41%) and pectin (36%) counterparts after 150 cycles. Impressively, the Si anode with DNB exhibits a much higher initial Coulombic efficiency (88.84%) than the PAA and pectin counterparts (87.68 and 76.85%, respectively), as shown in Fig. 5a. In terms of the average Coulomb efficiency, the DNB (99.6%) is also higher than that of PAA (99.3%) and pectin (99.4%), mainly ascribed to a stabile SEI containing Li_3_N. In addition, the long-term cycling performance of Si/Li half cells with different binders at a current density of 4.2 A g^−1^ (1 C) is also measured (Fig. 5c). The capacity in DNB-based cells can still maintain 1115 mAh g^−1^ after 300 cycles, much higher than those of the PAA (76.9 mAh g^−1^) and pectin (21.3 mAh g^−1^) counterparts. Furthermore, the Si electrode with DNB also delivers enhanced rate performance [1588 mAh g^−1^ at a current density of 8.4 A g^−1^ (2 C), 728 mAh g^−1^ at a current density of 16.8 A g^−1^ (4 C)], compared with PAA and pectin counterparts (Fig. 5b).

To further confirm the utility of DNB, we evaluated the cycle performance of Si/Li half cells at a high Si mass loading (4.0 mg cm^−2^), and NCM811 (11 mg cm^−2^)/Si full batteries. Encouragingly, the DNB still renders stable cycling performance, achieving a capacity of 1770 mAh g^−1^ in Si/Li half cells after 50 cycles (Fig. 5d). More importantly, good cyclability of NCM811 /Si full cells can also be obtained, with a capacity retention of 86% at 0.1 C after 50 cycles, evidently outperforming that of the PAA counterpart (Fig. 5e). The superior cyclability and rate performance can be mainly ascribed to the mechanically stable Si electrode enabled by DNB, clearly verifying the rationality of DNB.

## Conclusion

In summary, an endotenon sheath-inspired robust DNB is developed to address the cycling instability of Si electrodes during cycling. The as-developed binder shows excellent adhesion, mechanical properties, and self-healing capabilities mainly benefited by its reversible supramolecular hybrid network. Noting that the DNB-based Si electrode delivers mediocre reduced modulus and hardness, implying that DNB not only can suppress the excessive volume expansion of Si electrodes during cycling by a decent mechanical strength, but also can dissipate stress from volume change of Si particles and realize self-healing of electrode films. Apart from these benefits, DNB can participate in forming a Li_3_N/LiF-rich SEI contributing to reversible lithiation/delithiation behaviors of Si electrodes. As expected, the DNB achieves mechanically more stable Si electrodes than traditional PAA and pectin binders. Resultantly, the unique chemistry of DNB renders superior electrochemical performance of Si/Li half cells and NCM811/Si full cells, even with a high loading. This work helps inspire the binder design and also provides a promising strategy to achieve long-life Si anode-assembled lithium batteries.

## Supplementary Information

Below is the link to the electronic supplementary material.

Supplementary file1 (PDF 914 kb)
