# Correction to: An endotenon sheath-inspired double-network binder enables superior cycling performance of silicon electrodes

**DOI:** 10.1007/s40820-022-00881-x

**Published:** 2022-06-29

**Authors:** Meifang Jiang, Pengzhou Mu, Huanrui Zhang, Tiantian Dong, Ben Tang, Huayu Qiu, Zhou Chen, Guanglei Cui

**Affiliations:** 1 Qingdao Industrial Energy Storage Research Institute, Qingdao Institute of Bioenergy and Bioprocess Technology, Chinese Academy of Sciences, No. 189 Songling Road, Qingdao, 266101 People’s Republic of China; 2 Key Laboratory of Marine Chemistry Theory and Technology Ministry of Education, College of Chemistry and Chemical Engineering, Ocean University of China, No. 238 Songling Road, Qingdao, 266100 People’s Republic of China; 3 University of Chinese Academy of Sciences, Beijing, 100190 People’s Republic of China

## Correction to: Nano-Micro Lett. (2022) 14:87 https://doi.org/10.1007/s40820-022-00833-5

The original version of this article unfortunately contained some mistakes.

1. The authors found that the data unit in Fig. 3a–f is wrong.

The corrected version of Fig. 3 is given below:

**Fig. 3:**
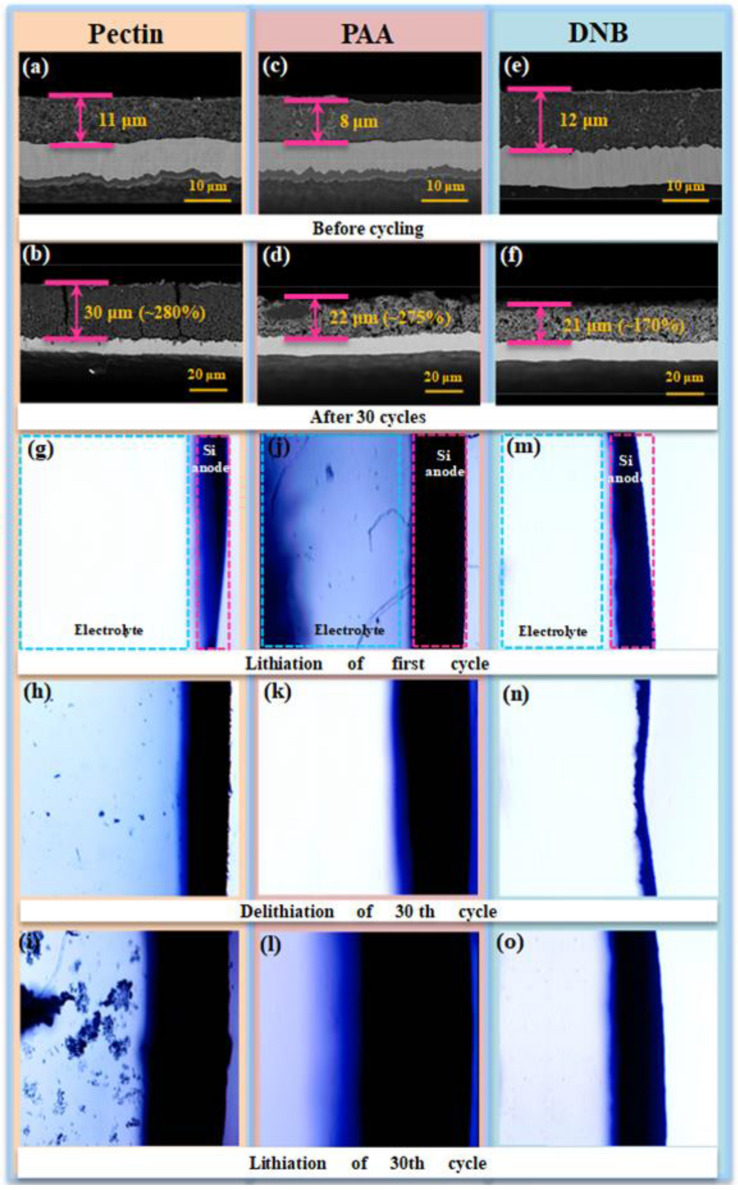
Cross-sectional SEM images of Si electrodes before (**a**–**c**) and after 30 cycles (**d**–**f**) with pectin, PAA, and DNB, respectively. In situ optical microscopy images of volume change of Si electrodes upon lithiation of first cycle and lithiation/delithiation of 30th cycle with (**g**–**i**) pectin binder, (**j**–**l**) PAA binder, and (**m**–**o**) DNB in assembled model cell module

2. The authors found that explanation of the data lines in Fig. 2e is wrong.

The corrected version of the explanation of Fig. 2e is given below:

The DNB can endure approximately 300% stretching and withstand stress up to about 1.5 MPa, as shown in Fig. 2e.

