# Supplementary material for: An Endotenon Sheath-Inspired Double-Network Binder Enables Superior Cycling Performance of Silicon Electrodes
Source: Nanomicro Lett. 2022 Apr 1;14:87. doi: 10.1007/s40820-022-00833-5 (PMC8975975; doi:10.1007/s40820-022-00833-5)
Supplement: Supplementary file 1 — Supplementary file1 (PDF 914 kb) [file 40820_2022_833_MOESM1_ESM.pdf]

Supporting Information for

An Endotenon Sheath-Inspired Double-Network Binder Enables Superior Cycling Performance of Silicon Electrodes

Meifang Jiang[1, 2, #], Pengzhou Mu[1, 3, #], Huanrui Zhang [1,*], Tiantian Dong[1, 2], Ben Tang[1], Huayu Qiu[1], Zhou Chen[1], Guanglei Cui [1,*]

[1] Qingdao Industrial Energy Storage Research Institute, Qingdao Institute of Bioenergy and Bioprocess Technology, Chinese Academy of Sciences, No. 189 Songling Road, Qingdao 266101, P. R. China

[2] Key Laboratory of Marine Chemistry Theory and Technology Ministry of Education, College of Chemistry and Chemical Engineering Ocean University of China, No. 238 Songling Road, Qingdao 266100, P. R. China

[3] University of Chinese Academy of Sciences, Beijing 100190, P. R. China

[#] Meifang Jiang and Pengzhou Mu contributed equally to this work.

*Corresponding authors. E-mail: zhanghr@qibebt.ac.cn (Huanrui Zhang); cuigl@qibebt.ac.cn (Guanglei Cui)

Supplementary Figures

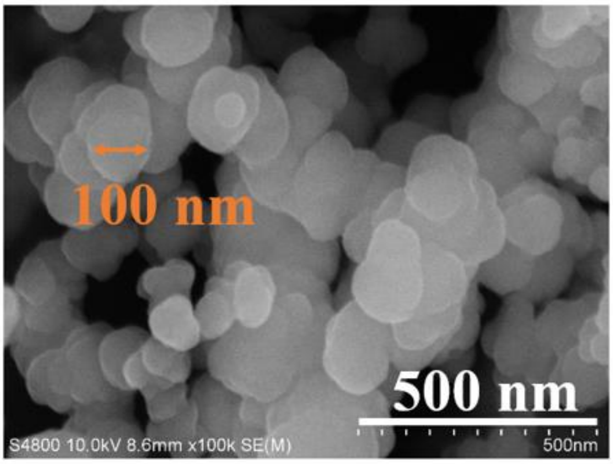

Fig. S1 Typical SEM image of silicon particles

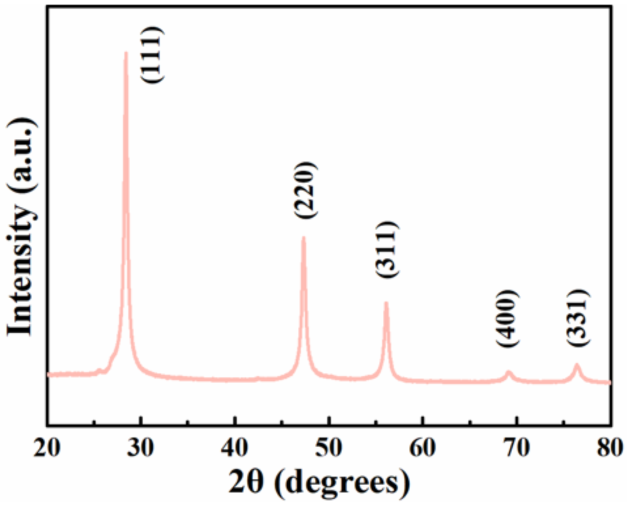

Fig. S2 XRD pattern of Si particles

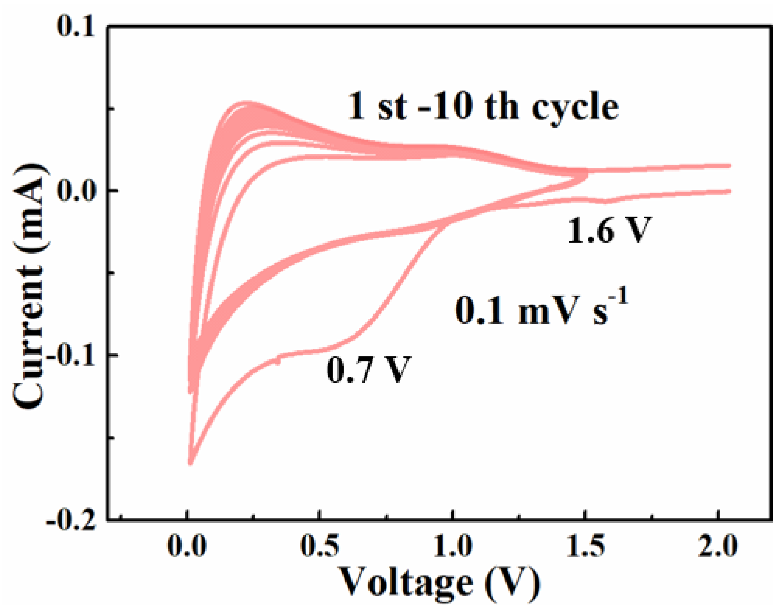

Fig. S3 CV curves of the DNB-based Si electrode at a scanning rate of 0.1 mV s^{-1}

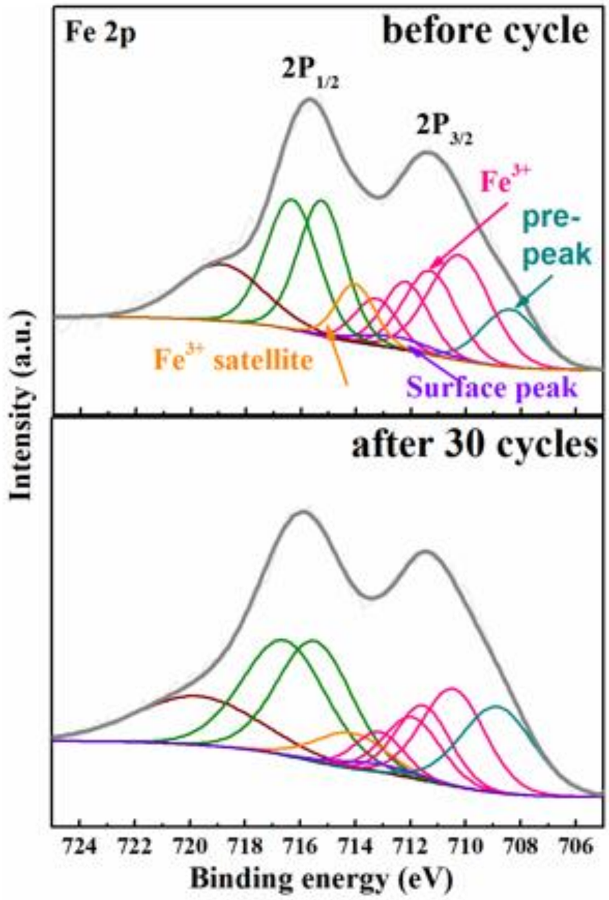

Fig. S4 Fe 2p XPS spectra with the DNB-based Si electrode before and after 30 cycles

Fe 2p XPS spectra of DNB-based Si electrode before and after 30 cycles is shown in Fig. S4 It can be seen that after battery cycling, there is no obvious peak change in the 2p3/2 band of Fe^{3+} that consists of four subpeaks at the range of 710~714 eV, and meanwhile no peak in the range of 706~708 eV corresponding to reduction products of Fe(0) or FeO. These findings indicate the good electrochemical stability of DNB.

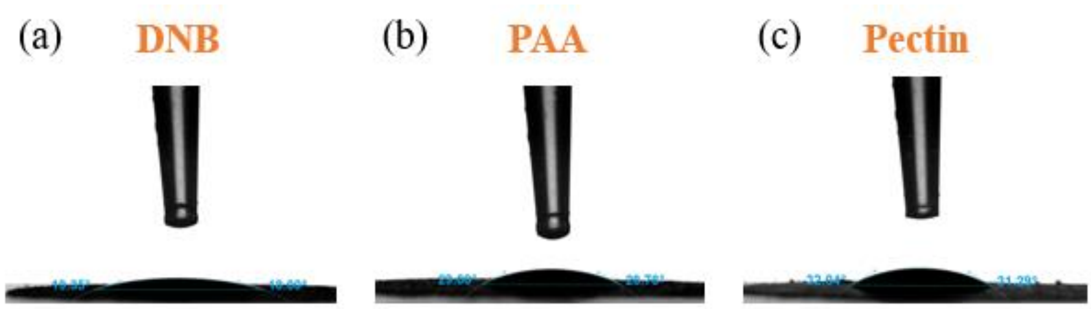

Fig. S5 Contact angles of $LiPF_6$/EC-DEC-FEC electrolyte on (**a**) DNB film (19.35°), (**b**) PAA film (29.00°) and (**c**) pectin film (32.04°)

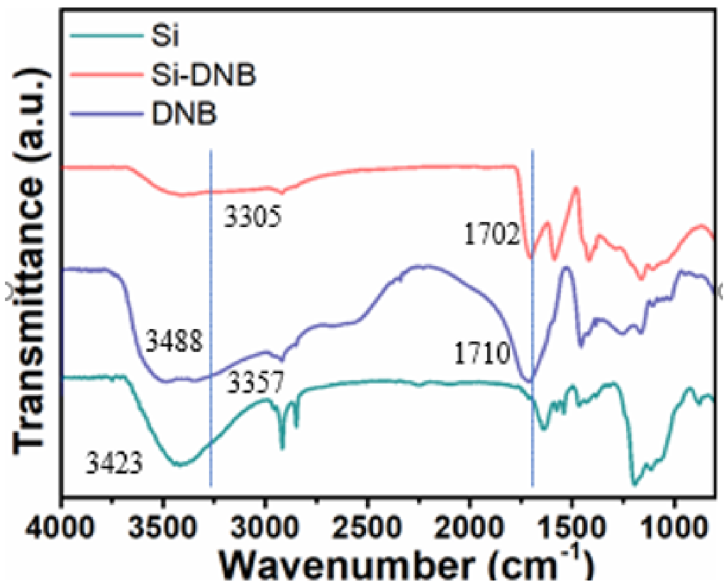

Fig. S6 FTIR spectra of Si, DNB and Si-DNB

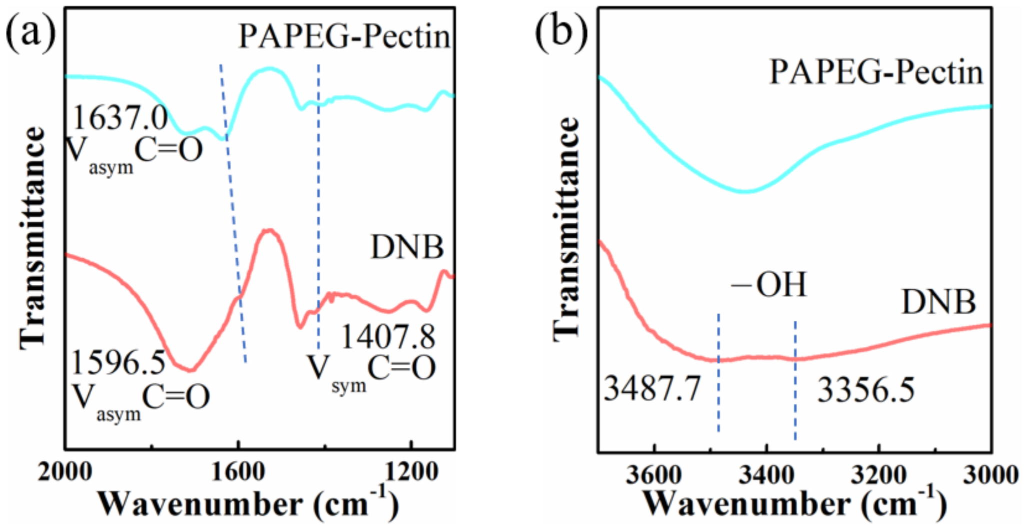

Fig. S7 FTIR spectra of DNB and PAPEG-pectin

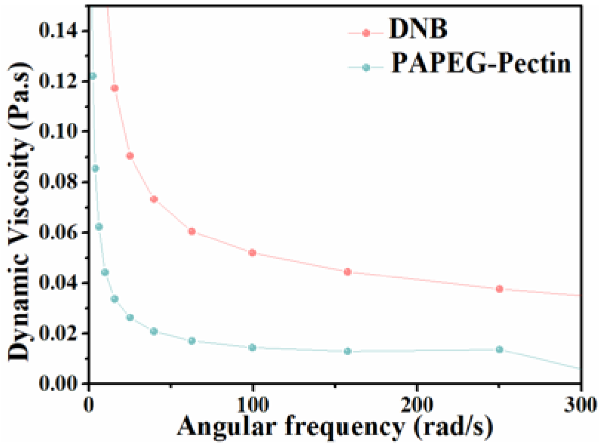

Fig. S8 Dynamic viscosity of DNB and PAPEG-pectin at different angular frequency and 17 °C

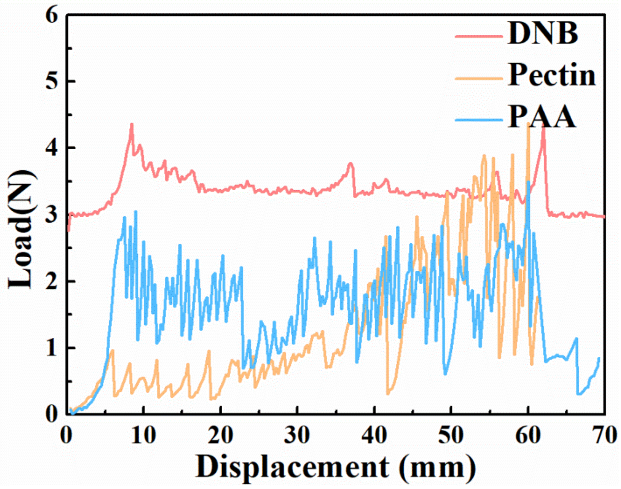

Fig. S9 Peel off test results of Si anodes based on DNB, PAA, and Pectin binders

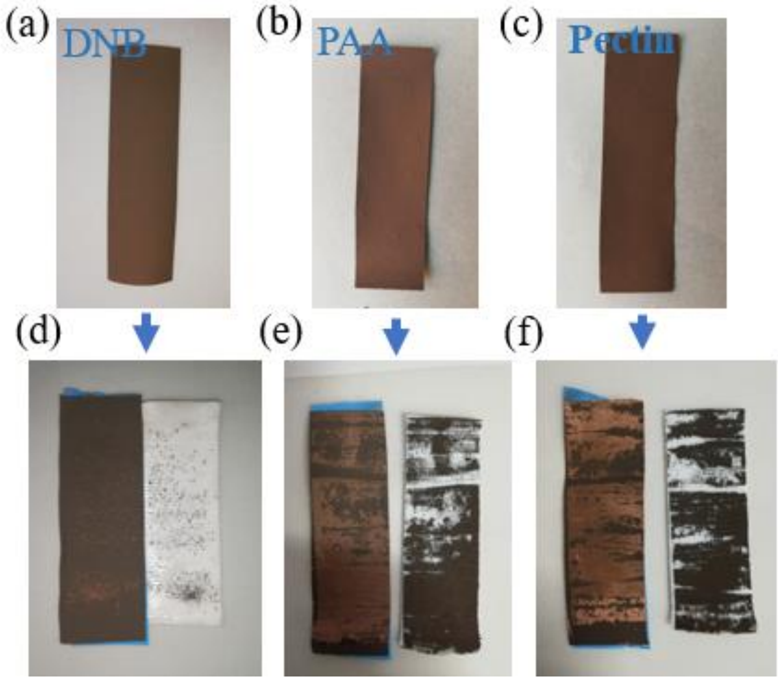

Fig. S10 Optical images of (**a, d**) DNB, (**b, e**) PAA and (**c, f**) pectin binder-based Si anodes before/after peeling test under the force of the tape

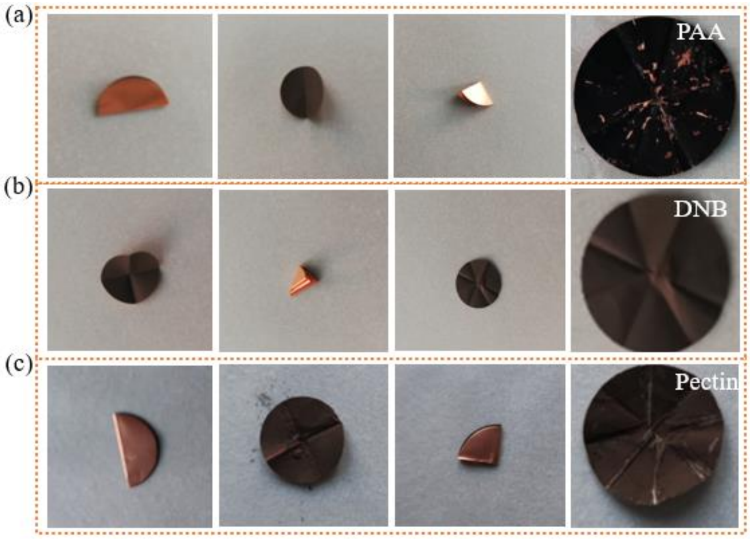

Fig. S11 Digital photographs of the (**a**) PAA, (**b**) DNB and (**c**) pectin-based Si electrode after being folded for one, two and three times, as well as their corresponding unfolded states

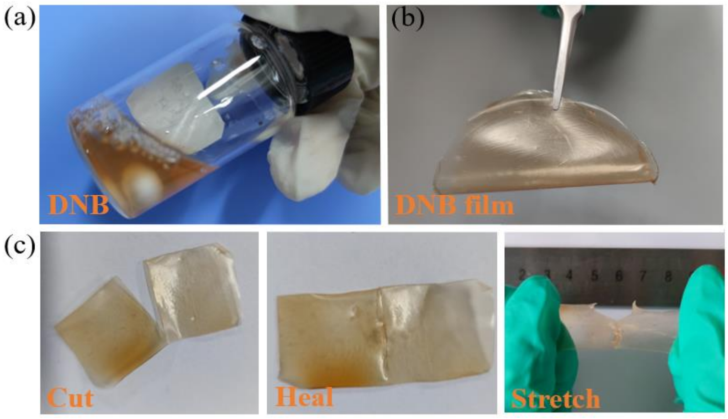

Fig. S12 The photographs of aqueous (**a**) DNB solution and (**b**) DNB film and (**c**) the self-healing ability of DNB

As shown in Fig. S12, the DNB film was prepared by coating the aqueous polymer-containing solution (5 wt%, Fig. S12a) on a Teflon mould at room temperature, followed by drying under a vacuum oven at 80 °C for 10 h. The dry polymer films were cut into 2 cm × 2cm for self-healing testing.

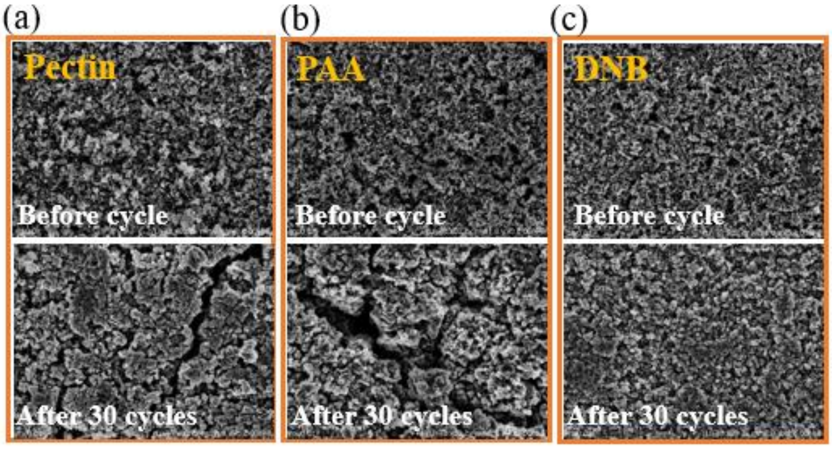

Fig. S13 Typical SEM images of (**a**) the pristine/the 30-cycled DNB-based Si electrode, (**b**) the pristine/the 30-cycled PAA-based Si electrode, (**c**) the pristine/the 30-cycled pectin-based Si electrode

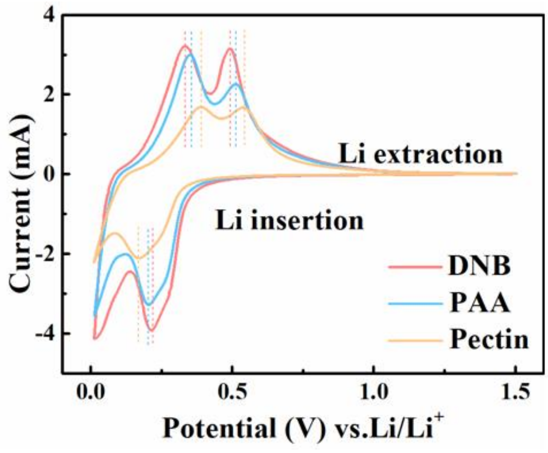

Fig. S14 CV curves of the different binders-based Si/Li cells at a scanning rate of 0.1 mV s^{-1} after five precycles

As illustrated in Fig. S14, the reduction peak at ~0.2 V is assigned to the reversible lithiation process of amorphous Si domains to Li-Si phase. The Si reduction peak at ~0.01 V is ascribed to the formation of amorphous Li–Si phases from the crystalline Si. In the delithiation process, two oxidation peaks at about 0.44 and 0.55 V are attributed to the delithiation process of Li-Si phases to amorphous Si domains. The higher peak current in the presence of the DNB indicates an overall increase in the dealloying and alloying kinetics.

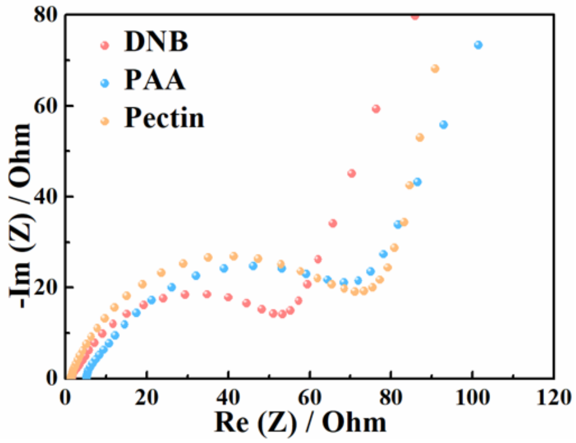

Fig. S15 Electrochemical impedance spectra of Si electrodes using DNB, as well as PAA and pectin binders after five precycles

As shown in Fig. S15, the DNB-based Si electrode has the smallest battery impedance (i.e., the sum of SEI impedance and charge-transfer resistance).

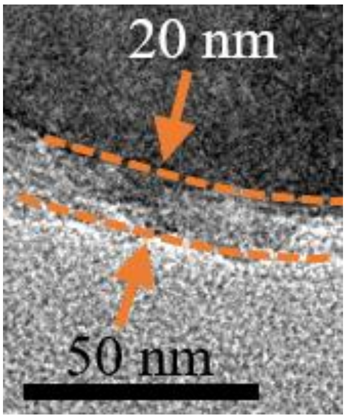

Fig. S16 Typical TEM image of the SEI on the Si surface with DNB after 30 cycles

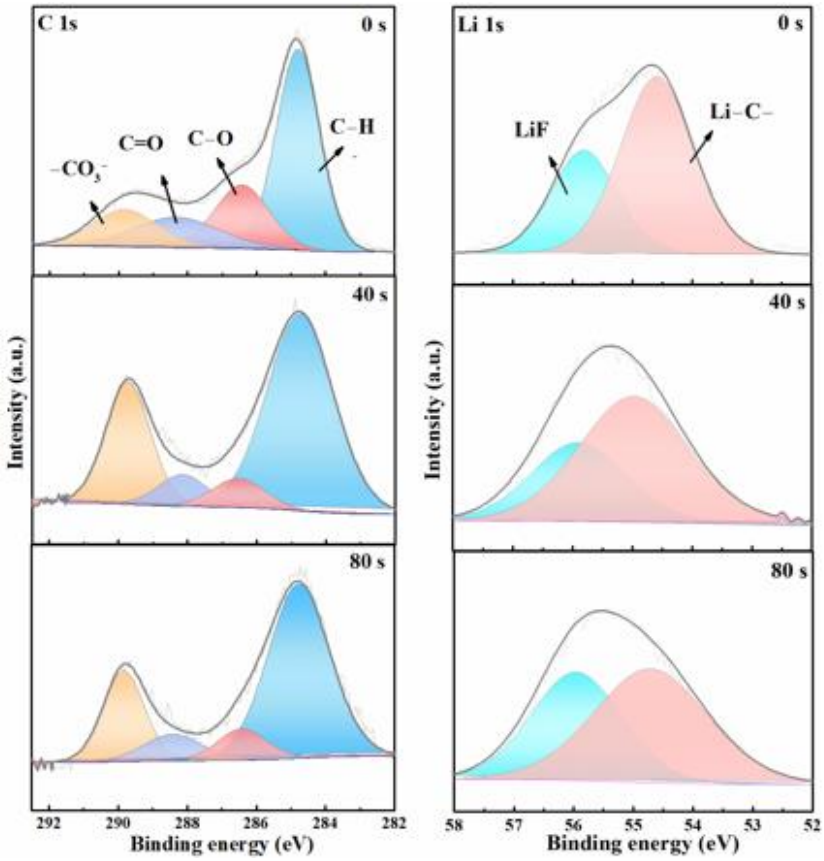

Fig. S17 (**a**) C 1s, (**b**) Li 1s XPS spectra of different Ar ion sputtering time with the PAA-based Si electrode after 30 cycles

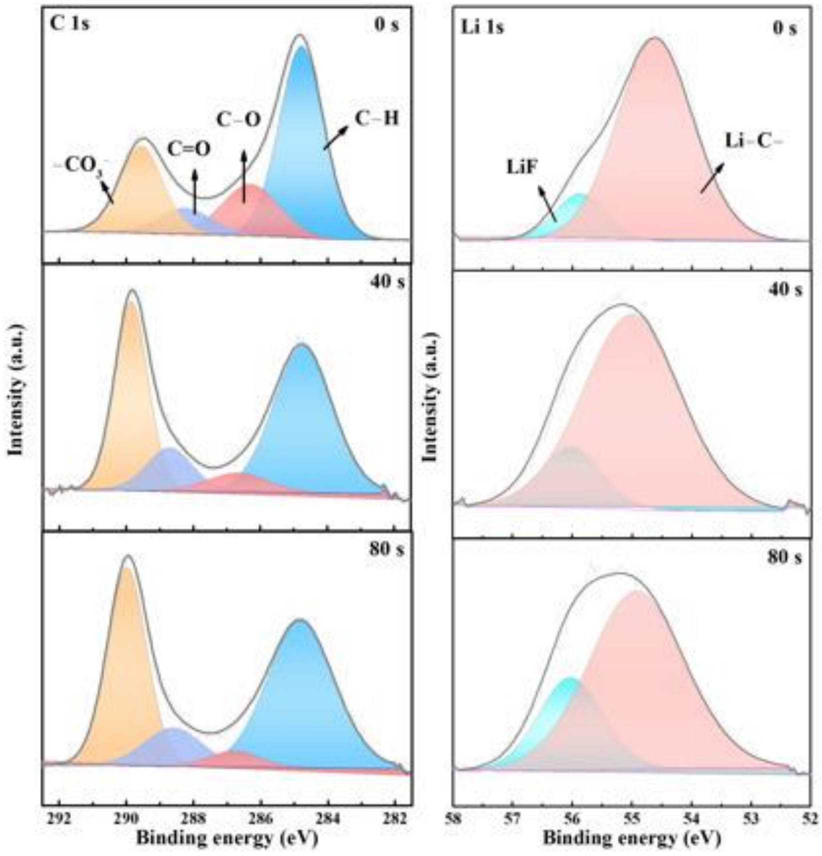

Fig. S18 (**a**) C 1s, (**b**) Li 1s XPS spectra of different Ar ion sputtering time with the pectin-based Si electrode after 30 cycles

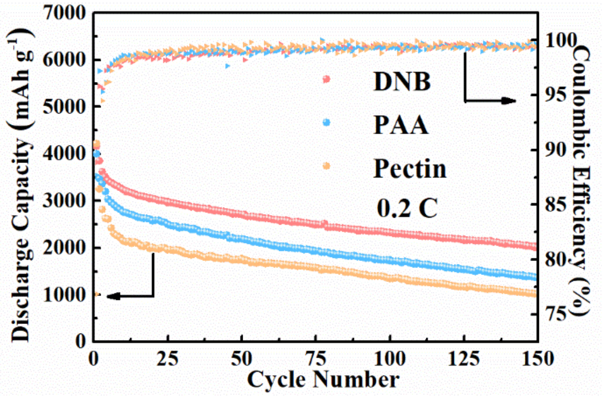

Fig. S19 Cycling performance and coulombic efficiency of the Si (0.93 mg/cm^2)/Li cells with different binders at 0.2 C, room temperature and a voltage range of 0.01-1.5 V
